# Why do ovigerous females approach courting males? Female preferences and sensory biases in a fiddler crab

**DOI:** 10.1002/ece3.2307

**Published:** 2016-07-10

**Authors:** Chun‐Chia Chou, Patricia R. Y. Backwell

**Affiliations:** ^1^ Research School of BiologyThe Australian National UniversityCanberraAustralian Capital Territory0200Australia

**Keywords:** Mating bias, origin of female preference, perceptual bias, *Uca mjoebergi*

## Abstract

Perceptual biases explain the origin and evolution of female preference in many species. Some responses that mediate mate choice, however, may have never been used in nonmating contexts. In the fiddler crab, *Uca mjoebergi*, mate‐searching females prefer faster wave rates and leading wave; however, it remains unclear whether such responses evolved in a mating context (i.e., the preference has effect on the fitness of the female and her offspring that arise from mating with a particular male) or a nonmating contexts (i.e., a female obtains direct benefits through selecting the male with a more detectable trait). Here, we compared the preferences of mate‐searching with those of ovigerous females that are searching for a burrow and do not concern about male “quality.” Results showed that as both mate‐searching and ovigerous females preferentially approached robotic males with faster wave rates. This suggests that wave rate increases detectability/locatability of males, but the mating preference for this trait is unlikely to evolve in the mating context (although it may currently function in mate choice), as it does not provide fitness‐related benefit to females or her offspring. Wave leadership, in contract, was attractive to mate‐searching females, but not ovigerous females, suggesting that female preference for leadership evolves because wave leadership conveys information about male quality. We provide not only an empirical evidence of sensory biases (in terms of the preference for faster wave), but the first experimental evidence that mating context can be the only selection force that mediates the evolution of male sexual traits and female preference (in terms of the preference for leading wave).

## Introduction

The origin of female mating preferences remains unclear in most animal species. Females of many species have perceptual biases that, while they currently influence mate choice, evolved in other contexts (Endler and Basolo 1998; Kokko et al. 2003; Ryan and Cummings 2013). Sensory systems are under selection in multiple contexts, and these selection pressures will constrain the types of mating signals and the evolution of female preferences (Endler 1992). There are many studies showing that perceptual and sensory systems evolved in a nonmating context, but now influence mating preferences and contribute to the evolution of male sexual traits are common (Endler and Basolo 1998; Kokko et al. 2003; Ryan and Cummings 2013). Through selecting more stimulating signals, females acquire direct benefits, such as reduction of predation risk (Nakano et al. 2014) or reduced searching costs (Ryan and Keddy‐Hector 1992), thereby maintaining the mating preferences. For example, the structure built by the male fiddler crab at the opening of burrow (e.g., hood and pillar) can help females to orientate the burrow and enhance male attractiveness (Christy 2007). The idea of perceptual biases originating in nonmating contexts is now so well established, that it is easy to assume that all mating biases are derived from responses in other contexts. However, there are some traits that mediate mate choice may have never been used in nonmating contexts (Christy 1995), yet the idea is less explored.

A widespread pattern in mate choice studies is that, when there is a directional preference, females prefer males with brighter, faster, louder, or more exaggerated signals (Endler 1992). This is true in many species of fiddler crabs; for example, in banana fiddler crab, females prefer faster wave rates and leading waves (Backwell et al. 1998; Reaney et al. 2008; Callander et al. 2012; Kahn et al. 2014). It is possible that these two male traits enhance male detectability and locatability that help to reduce predation risk on females when they sample potential mates. Mate‐searching females visit several males before selecting a mate (over 100 visits in some species: Derivera 2005). It is dangerous for mate‐searching females to move around on the mudflat, visiting potential mates. Therefore, the faster they can localize and approach a courting male, the less time they will be subjected to potential predation (and other costs).

Perceptual biases that increase the detectability or locatability of males are common, but an alternative mechanism to explain the origin of mating preference is that exaggerated courtship displays (e.g., faster wave rates and leading waves) infer higher male “quality” (MØller 1990; Wilkinson et al. 1998). Sexual displays are generally metabolically demanding (MØller et al. 1998; Basolo and Alcaraz 2003; Allen and Levinton 2007), and may result in decreased foraging time (Andersson 1994; Cowles and Gibson 2015). In fiddler crabs of the genus *Uca*, males have greatly enlarged one of their feeding claws and use it as a weapon and wave it to attract mate‐searching females. The major claw has lost it feeding function (Levinton et al. 1995) and adds a considerable physiological cost (Matsumasa and Murai 2005; Allen and Levinton 2007). Faster wave rates and the production of leading waves may advertise the ability of the male to bear these costs (Murai et al. 2009).

**Figure 1:**
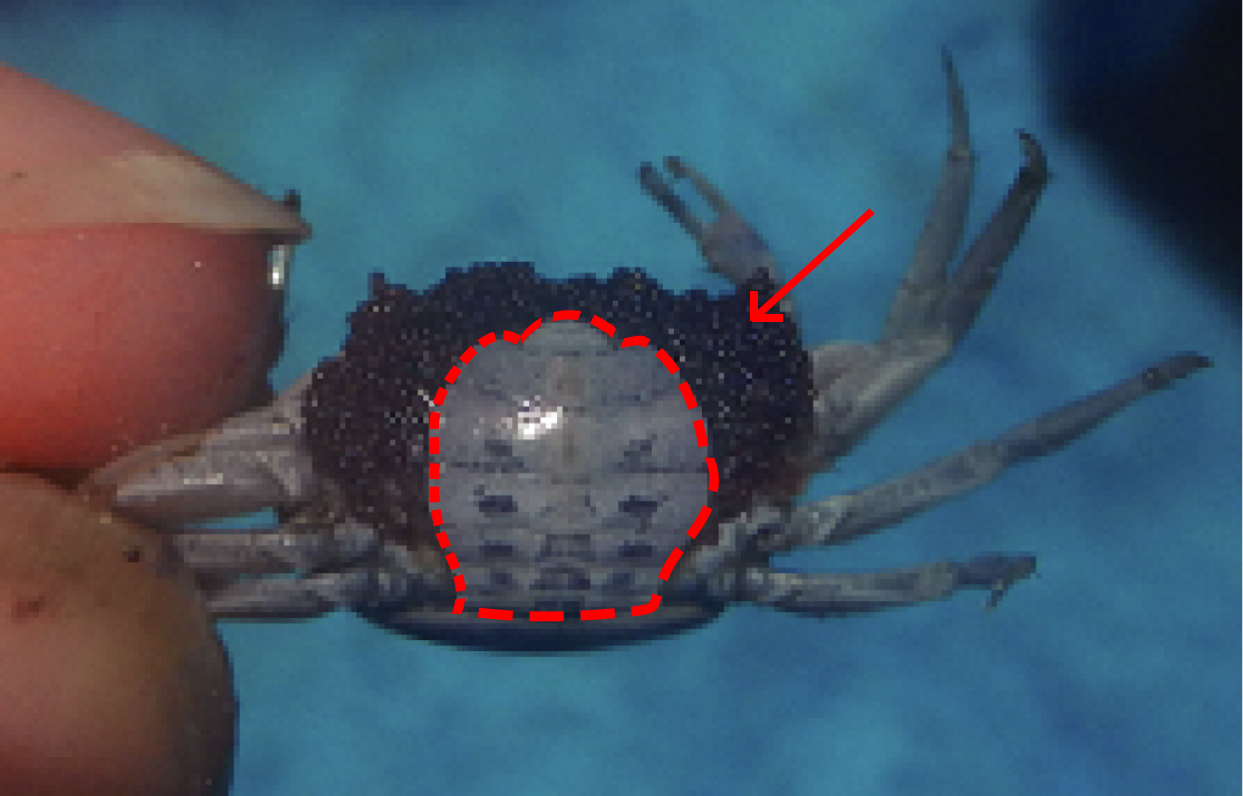
An ovigerous female of banana fiddler crabs. It has a clutch of fertilized eggs (red arrow) in its abdominal cavity, blocking access to its gonopore. The frame circled by the red line is an abdominal flap.

Here, we compare mating preferences (for faster wave rates and leading waves, respectively) of mate‐searching and ovigerous females in banana fiddler crabs, *Uca mjoebergi*, for elucidating the origin of mating preferences. In order to ensure that non‐mate‐searching females are definitely not interested in the quality of the male, we use ovigerous females. Ovigerous females (Fig. 1) would normally be underground, incubating their clutch and are not seeking for mates. Occasionally, they are evicted from their burrows and need to move around the population for searching for an empty burrow in which to continue their incubation. They approach males successively and walk up to their burrows before moving on in their search for an empty burrow. Why do ovigerous females approach courting males? Rather than being concerned with the quality of the males, the most likely explanation is that the female is ensuring that she always has a path integration map to a nearby burrow in order to lower her predation risk (Christy 2007). In the event of a predator attack, the female will be able to quickly and directly approach the burrow and temporarily hide in its entrance (Christy 2007).

If ovigerous females also preferentially approach males with fast, leading waves, it would suggest that the preference evolved in the nonmating context (i.e., a female obtains direct benefits through selecting the male with a more detectable trait). However, if ovigerous females do *not* have these preferences, it would suggest that these traits (and the preference for them) may have evolved in the context of mate choice (i.e., a female and her offspring obtain fitness‐related benefits through mating with the particular male) and may give females information about male “quality.” We also test the female preferences for fast and leading waves presented at different distances. We predict that mate searchers will travel further to get the more preferred male, but ovigerous females will always prefer the nearer male.

## Methods

### Study species

The banana fiddler, *Uca mjoebergi*, is a small crab (carapace width ± 2 cm) that lives in large, high‐density, mixed‐sex populations on intertidal mudflats. Each individual (both male and female) defends a territory with a central burrow that is used as a water source, a mating and incubation site, a heat sink, and a refuge to escape from predators. The immediate small area (±10‐cm diameter) surrounding the burrow is used for feeding and courting. Mating occurs over 5 days for each 14‐day tidal cycle. The amplitudes of the semidiurnal tides ranged from 1.49 to 6.68 m during the study. A female that is ready to mate will leave her territory and wander through a subset of the population of waving males, visiting 1–6 males (Reaney and Backwell 2007) and briefly entering their burrows, before selecting a mate. Mating occurs inside the male's burrow, and he then guards the female for 1–4 days until she extrudes her clutch of eggs onto her pleopods. After extrusion of the eggs, the female can no longer re‐mate, and at this time, the male leaves and re‐seals her in the burrow, where she remains incubating for the following 16–20 days. After incubation, the female re‐emerges at nocturnal spring tide and release her pelagic larvae into the water.

### Robotic crab trials

We studied a population of banana fiddler crabs at East Point Reserve, Darwin, Australia (12°24′32″S; 130°49′50″E), in October‐November 2015. We used two types of females in this study: mate‐searching females and ovigerous females. Both types of females were found while they moved through the population of courting males. They are easily distinguishable as ovigerous females have a clutch of eggs protruding from their abdominal flaps (Fig. 1). We caught 20 mate‐searching and 20 ovigerous females, measured their carapace widths, and tested their mating preferences using robotic crab models (Booksmythe et al. 2008; Reaney 2009; Callander et al. 2012). The custom‐built robots are run from a central control box that decodes sound files transmitted from a portable CD player. Each robot is encoded by a unique frequency, and relay switches in the control box supply current to the appropriate robot to activate a wave. The robotic crab units have an internal two‐cam system (GWServo S03N 2bb) that controls the movement of a metal arm that exactly mimics the male courtship wave. The motor is enclosed in a plastic container that we bury in the sediment so that only the metal arm protrudes. A replica claw is attached to the arm. It is constructed from Hydrostone in a latex mold of a real claw, and painted with Dulux model paint that falls within the natural color range (spectrometry: Detto et al. 2006).

The female was placed under a small, inverted transparent cup on the choice arena. The two robotic crabs were placed 5 cm apart on the same side of the arena, 20 cm away from the female, and directly facing her. Each had an identical claw replica that was 20.2 mm long. In the first trial, the female was presented with the choice between a fast and a slow wave rate (16.8 or 4.2 waves/min). The waves were synchronous every fourth wave, and then, the “slow” robot missed three waves. In the second trial, the female was presented with a leading and following wave: both robots waved at the same rate (16.8 waves/min), but the follower's wave started 0.9 s after the leader's wave (a quarter the duration of the wave).

In trials 3, 4, and 5, one robot was placed at 20 cm and the other at 50 cm away from the female release point. In trial 3, both robots produced identical, synchronous waves at the fast wave rate (16.8 waves/min). In trial 4, the further robot had a fast wave rate (16.8 waves/min) and the closer robot had a slow wave rate (4.2 waves/min). In trial 5, the further robot produced a leading wave and the closer robot produced a wave that followed it by 0.9 sec. Trials 6, 7, and 8 were identical to 3, 4, and 5, but the distances used were 10 and 20 cm.

The female was remotely released after she had been presented with three full waves from each of the robots (±2 min). A positive response (i.e., female choice) was scored if the female moved toward one robot and even touched the rim of the unit. We eliminated trials in which the female dashed, moved to the edge of the arena, or sat still for >5 min. Each female was tested once in each of five experiments (i.e., trial 1–5 or trial 1–2 and 6–8). As females naturally approach and leave up to six males before selecting a mate, it is reasonable to test them in consecutive experiments (Reaney and Backwell 2007). All females were returned to the natural habitat after taking part in the experimental trials. The order of trials, positions of robots, and position of stimuli were alternated between females. We tested the female responses using binomial tests in SPSS 17.0.

## Results

There was no difference in the size of mate‐searching and ovigerous females (mate‐searching females: $\overset{¯}{x}$ = 8.99, SD = 0.98, *n* = 54; ovigerous females: $\overset{¯}{x}$ = 8.99, SD = 1.05, *n* = 45; independent samples *t*‐test *t* = 0.005, df = 91, *P*_*2*_ = 0.99).

Table 1 gives the choices made by mate‐searching and ovigerous females and the binomial *P* values. As there were 16 binomial tests, we adjusted the *α* level using the Benjamini–Hochberg False Discovery Rate test with a false discovery rate of 15%. The adjusted *α* levels are given in Table 1, and the significance of the *P* values relative to the adjusted *α* are given.

**Table 1:** Mate choice of mate‐searching and ovigerous females

Trial distance (cm) and wave		Mate‐searching females				Ovigerous females			
		*N*		Binomial *P*	FDR‐adjusted *α*	*N*		Binomial *P*	FDR‐adjusted *α*
20 fast	20 slow	35	9	**0.000**	0.009	24	10	**0.024**	0.056
20 lead	20 follow	40	20	**0.013**	0.047	21	13	0.229	0.075
50 fast	20 fast	4	21	**0.001**	0.019	0	20	**0.000**	0.009
50 fast	20 slow	6	14	0.115	0.066	1	19	**0.000**	0.009
50 lead	20 follow	5	22	**0.002**	0.028	2	16	**0.001**	0.019
20 fast	10 fast	4	17	**0.007**	0.038	6	14	0.115	0.066
20 fast	10 slow	19	3	**0.001**	0.019	8	12	0.503	0.084
20 lead	10 follow	12	10	0.832	0.094	3	17	**0.001**	0.019

The *α* levels were adjusted by Benjamini–Hochberg False Discovery Rate test with a false discovery rate of 15%. Significant False Discovery Rate (FDR)‐adjusted *P* values are in bold if they are significant.

*Mate‐searching females* preferred the faster wave rate and leading waves when two robotic males were presented at equal distance away from the female. When the two waves were synchronous and presented at the same rate, mate‐searching females preferred the closer robotic male, both when the differences in distance was large (20 cm vs. 50 cm) or small (10 cm vs. 20 cm). They showed no preference for fast waves when they were required to travel an additional 30 cm in order to reach them (slow males at 20 cm, fast males at 50 cm), but they preferred faster waving males when the difference in distance was small (slow males at 10 cm, fast males at 20 cm). In contrast, mate‐searching females showed no preference for a leading over a following wave when the difference in distance was small (follower at 10 cm, leader at 20 cm), but significantly preferred the closer male (even though he produced following waves) when the difference in distance was greater (follower males at 20 cm, leaders at 50 cm.

*Ovigerous females*, when presented with equidistant males, preferred the faster waving male, but had no preference for leading waves. When ovigerous females were presented with stimuli that were 30 cm apart, they significantly preferred the closer stimulus when (1) the stimuli were identical (synchronous, fast wave rate at 20 cm and at 50 cm); (2) the stimuli differed in wave rate (fast rate at 50 cm, slow rate at 20 cm); and the stimuli differed in wave timing (leader at 50 cm, follower at 20 cm). When the difference in distance was small (10 cm vs. 20 cm), ovigerous females showed no preference for either stimulus when they were identical (synchronous, fast wave rate at 10 cm and at 20 cm), or when they differed in wave rate (fast rate at 20 cm, slow rate at 10 cm). They significantly preferred the closer robot when they differed in wave timing (leader at 20 cm, follower at 10 cm).

When there was a difference in the distances of the two robots, ovigerous females either strongly preferred the nearer robot or (when the difference was small) showed no preference for either. They never preferred the robot in the farther distant.

## Discussion

Ovigerous females are not seeking for mates. They move around on the mudflat in order to find a new burrow and, like mate‐searching females, they successively visit and leave the burrows of multiple males. This behavior ensures that the female always has a kinesthetic map to the last‐visited burrow, and she can return quickly and directly to it when threatened by a predator (Christy et al. 2002; Zeil and Layne 2002). Ovigerous females should not be concerned with the quality of the males they approach. Here, we show that ovigerous females preferentially approached robotic crabs that waved at a faster rate, but had no preference for those that produced leading waves. In contrast, mate‐searching females showed a preference for both faster waves rates and leading waves. Fast wave rate is clearly a trait that is attractive to both mate‐searching and burrow‐searching/ovigerous females. As ovigerous females are not concerned with the “quality” of the male they approach, we suggest that faster wave rate is attractive to them because it increases the visibility and locatability of the male. Mate‐searching females would also be more likely to approach faster waving males, either due to their increased visibility/locatability or due to a possible correlation between wave rate and male “quality.”

In contrast, wave leadership is a trait that is attractive to mate‐searching females, but of no interest to nonmate searchers. This suggests that leadership may (1) convey some kind of information about the quality of the male that produces it and (2) leadership does not increase a male's detectability or locatability. It appears unlikely that wave leadership evolved in a nonmating context, but wave rate may have as it influences all females that move across the mudflat.

All females, whether mate searching or not, should preferentially approach the nearer of two identical stimuli due to the decrease in travel time, energy expenditure, and predation risk (Milinski and Bakker 1992; Wikelski et al. 2001; Byers et al. 2005; Booksmythe et al. 2008). The ovigerous females that we tested either chose the closer male or had no preference when the difference in distance was small. The mate‐searching females preferentially approached the nearer stimulus when they were identical, but their strong preferences for fast wave rates and leading waves obscured their tendency to approach the nearer signal. The interactions between female preferences for wave rates, leadership, and distance require further testing for mate‐searching females, but the data presented here are sufficient to demonstrate that the two male traits examined (wave rate and wave leadership) did not alter the preference that ovigerous females had for approaching the nearer of the two signals; but that fast wave rate overturned the preference that mate‐searching females had for the 10‐cm signal over the 20‐cm signal.

The present study concentrated on elucidating the origins of mating preference for faster waves and leading waves (i.e., evolves in mating or nonmating context). One possible direction for further studies is to provide evidence of accounting for the direct benefits in terms of the increased detectability and locatability. For example, in order to test whether the preference for the pillar built by male fiddler crabs originated in the context of predation, Kim et al. (2007, 2009) manipulated predation pressure and found that female fiddler crabs were more attracted by the male building pillar near his burrow under escalated predation risk. Additionally, it is also likely to experimentally discriminate the selection on the sexual signal per se and the signal intensity through manipulating predation pressure. For example, in *Uca lacteal*, females preferred males with full‐size semidome only under escalated predation risk, suggesting that the selection on the size of semidome (signal intensity) is independent of the selection on the semidome (Zhu et al. 2012).

## Conclusion

All females have sensory systems with perceptual biases. It is easy to think of these biases as originating and evolving to allow the female to find food or avoid predators and that, at some later stage, the biases form the basis of a signaling system allowing the females to detect, locate, and choose mates. There is overwhelming evidence that this occurs (Ryan and Keddy‐Hector 1992; Christy 1995; Endler and Basolo 1998; Kolm et al. 2012; Ryan and Cummings 2013), but we should not lose sight of the cases where female perceptual biases may have evolved in the context of mate choice. The common pattern of directional female mating preferences for larger, louder, brighter signals (Endler 1992) is often explained by the reduced search costs or greater sensory stimulation that such signals provide (Ryan and Keddy‐Hector 1992). Again, this is often true (Endler 1992; Stuart‐Fox et al. 2007), but it should not be assumed. Here, we show that faster wave rates attract females that are searching for a mate or for a burrow, suggesting that faster wave rates increase the detectability or locatability of males in all contexts. This trait is likely to provide greater sensory stimulation to females and may have evolved in a nonmating context (a direct, nonrelated to fitness, fitness that a female and her offspring obtain although selecting a male with a more detectable trait). Wave leadership, in contrast, does not attract non‐mate‐searching females and so is unlikely to decrease search costs or provide greater sensory stimulation to females that are not searching for a mate. It is more likely, in this particular case, that the signal content, rather than the signal efficacy, is important.

## Conflict of Interest

We declare no conflict of interest.
